# Carbonaceous Particulate Matter Emitted from a Pellet-Fired Biomass Boiler

**DOI:** 10.3390/atmos10090536

**Published:** 2019-09-11

**Authors:** Michael D. Hays, John Kinsey, Ingrid George, William Preston, Carl Singer, Bakul Patel

**Affiliations:** 1 Office of Research and Development, U.S. Environmental Protection Agency, Washington, DC 20460, USA; 2 JACOBS Inc./CSS, 1910 Sedwick Road, Durham, NC 27713, USA; 3 Senior Environmental Employment (SEE) Program, Washington, DC 20460, USA

**Keywords:** particle matter, SVOCs, GC–MS, PAH, pellets

## Abstract

Biomass pellets are a source of renewable energy; although, the air pollution and exposure risks posed by the emissions from burning pellets in biomass boilers (BBs) are uncertain. The present study examines the organic species in fine particle matter (PM) emissions from an BB firing switchgrass (SwG) and hardwood (HW) biomass pellets using different test cycles. The organic and elemental carbon (OC and EC) content and select semivolatile organic compounds (SVOCs) in filter-collected PM were identified and quantified using thermal-optical analysis and gas chromatography–mass spectrometry (GC–MS), respectively. Fine PM emissions from the BB ranged from 0.4 g/kg to 2.91 g/kg of pellets burned of which 40% ± 17% *w/w* was carbon. The sum of GC–MS quantified SVOCs in the PM emissions varied from 0.13 to 0.41 g/g OC. Relatively high levels of oxygenated compounds were observed in the PM emissions, and the most predominant individual SVOC constituent was levoglucosan (12.5–320 mg/g OC). The effect of boiler test cycle on emissions was generally greater than the effect due to pellet fuel type. Organic matter emissions increased at lower loads, owing to less than optimal combustion performance. Compared with other types of residential wood combustion studies, pellet burning in the current BB lowered PM emissions by nearly an order of magnitude. PM emitted from burning pellets in boilers tested across multiple studies also contains comparatively less carbon; however, the toxic polycyclic aromatic hydrocarbons (PAH) in the PM tested across these pellet-burning studies varied substantially, and produced 2–10 times more benzo[*k*]fluoranthene, dibenz[*a,h*]anthracene and indeno[*1,2,3-c,d*]pyrene on average. These results suggest that further toxicological evaluation of biomass pellet burning emissions is required to properly understand the risks posed.

## 1. Introduction

Interest in alternative and renewable energy sources is growing worldwide due, partly, to the economic, security, and environmental concerns associated with burning fossil fuels. Renewable energy accounts for ~13% of total U.S. and world energy supplies currently [1]. Data for 2017 show that approximately 5% of the total renewable energy supply in the U.S. (11.6 quadrillion kJ) is used in the residential sector, and that biomass in the form of wood or wood byproducts dominates renewable energy consumption for residences [2]. In certain European and U.S. regions, the use of biomass boilers (BBs) and furnaces, which are also sometimes referred to as hydronic heaters, is increasing [3]. Compared with most residential biomass burning appliances, BBs have unique operating characteristics, large fire-box enclosures capable of burning multiple fuel types, and relatively low emissions stack heights [4]. However, these latter two characteristics of BBs create serious concern about air pollution and exposure risk [5]. Past studies have observed high levels of polycyclic aromatic hydrocarbons (PAH), polychlorinated compounds, heavy metals, and multiple additional biologically antagonistic chemical species in BB emissions and ashes [6].

Although producing biomass pellets requires substantial capital investment, burning pellets may offer several advantages, including lowering certain toxic pollutant emissions [4]. Biomass pellets can be produced from a variety of indigenous forest or agricultural vegetation. Their composition can be mixed and their properties engineered and homogenized to (i) simplify storage and transport, (ii) streamline introduction to different furnace and boiler technologies, and (iii) optimize combustion conditions [7]. Despite the many advantages, relatively few studies examine the organic chemical species in fine particle matter (PM) emissions from BBs burning biomass pellets [8-11]. In comparison, the present study is unique in that it investigates the organic chemical species in emissions while simulating real-world BB operation. These emissions are then examined relative to fixed, steady-state BB operations at low and nominal loads. To our knowledge, the current research is also the first to compare BB emissions with those from different residential biomass burning appliances while emphasizing the toxicological PAH compounds.

Such emissions data are critical to developing new source performance standards and reliable future emissions inventories. Emissions inventory data propagate into the global climate, chemical mass balance, and atmospheric dispersion models used to develop scientifically sound air quality and environmental regulatory policy. The present study quantitatively evaluates PM, organic and elemental carbon, and semivolatile organic compound (SVOC) emissions from the BB burning both switchgrass (SwG) and forest hardwood pellets (HW). Compared to other biomass sources, grass pellets have high energy conversion efficiency and are economically feasible, and grass farming has land use and pesticide and nutrient management benefits. HW pellets produce less ash and clinker, contain less chloride, produce less NOx and SOx emissions, and are generally ranked higher for large-scale applications [12]. The current study finds that the biomass pellet type produces different SVOC concentration distributions, but only a has minor influence on PM and organic and elemental carbon emissions; the effect due to test cycle is the greatest on these emissions. In the final analysis, compared with wood burning, pellet burning produces substantially different emissions of PAH, a result that suggests further toxicological evaluation of pellet burning emissions is required.

## 2. Experimental

All biomass pellet combustion tests using the BB were performed at the U.S. Environmental Protection Agency’s Research Triangle Park, North Carolina campus. The BB unit was housed outside the facility during the experiments. A schematic of the BB testing facility and BB photo are presented in the supplementary information.

### 2.1. Pellet Fuels

Both HW and SwG pellets were tested in the current study. The HW pellets (Fiber Energy Products, LLC, Mountain View, AZ) were certified as premium grade by the Pellet Fuel Institute (PFI). The SwG pellets were provided by SwitchGreen, Kingston, Ontario. Both pellet types were supplied in 18 kg plastic bags and underwent proximate, ultimate, and ash mineral analyses prior to testing See Table S1 in supplementary material for results.

### 2.2. Pellet-Burning Biomass Boiler (BB)

The pellet fuels were burned in the BB unit (Reka, model HKRST/V-FSK20, Aars, Denmark). The unit was an open-grate, fire tube boiler with a nominal output of 16,500 kCal (72 MJ or 20 kW). It is capable of burning multiple fuel types. Pellet fuel was introduced to moving step-grates with an automated feed screw as air was directed under and over the fuel bed during burning. The heat was transferred using a two-pass steel-plate heat exchanger bearing 50 mm fire tubes. The tubes were cleared by back-flushing with compressed air, producing high transient PM emissions. Water was circulated from the BB through a heat exchange loop for simulation of residential heating as described previously [4]. To meet heat load demand requirements, the fuel feed (4.2 kg/h maximum) was controlled digitally using stack temperature and oxygen sensors. Three heat load demand profiles were used in the present study: (i) steady-state operation at 100% load (72 MJ/h), (ii) steady-state operation at 25% load (18 MJ/h), and (iii) the Syracuse cycle with a maximum heat load of ~36 MJ/h. The Syracuse cycle was a diurnal heat demand profile for a typical 232 m^2^ home in Syracuse, NY in January. All test cycles were compressed into 6 h sampling periods. Replicate tests (*N* ≥ 2) were performed for each load demand condition and fuel type. Full load testing with HW pellets were run in triplicate, and *n* = 4 for low load testing with SwG pellets. Note that the unit failed to operate in the <15% load category required of EPA’s M28WHH certification test. Thus, the unit is impractical for heating in the U.S. market without thermal storage. Presently, wood pellets are produced primarily in North America and exported to northern European nations.

### 2.3. PM Emissions Sampling

Stack and dilution sampling methods were conducted in accordance with ASTM method E2515-11 and EPA Method 1A. Briefly, the BB stack emissions were directed into a conical hood connected to a 12 m long, 0.25 m diameter dilution duct that achieved a dilution ratio of approximately 15:1. Boiler emissions were extracted from the dilution duct using a vertically positioned sampling probe. The probe flow was directed to a multi-filter sampling array that collected PM using 47 mm diameter Teflon and pre-fired (550 °C, 12 h) quartz fiber filters. PM mass on the Teflon filters was determined using an established gravimetric analysis procedure [13]. A quartz fiber filter was also placed behind the Teflon one for estimating the positive adsorption artifact as described by Subramanian et al. [14]. Characterization of the carbonaceous aerosol was the focus of this study. For this purpose, a total of sixty-one quartz filters were collected across the test matrix. Filter samples were stored at −65 °C prior to further chemical analyses. An electrical low-pressure impactor indicated that all particles emitted from the BB were <1 μm in aerodynamic diameter.

### 2.4. Organic and Elemental Carbon

The organic and elemental carbon (OC and EC) composition on each PM filter (1.5 cm^2^ punch size) was measured using thermal-optical analysis and a modified NIOSH 5040 method [15]. This bulk chemical analysis approach was used to estimate the total extractable OC on the filter. Past studies have demonstrated that at least 100 μg of filter OC is required to achieve reasonable gas chromatography–mass spectrometry (GC–MS) results. To ensure adequate OC mass was available for GC–MS analysis of the BB PM emissions, a sample compositing strategy was applied. Sample composite loads (*n* = 3 filters) ranged from 37 μg to 1455 μg. On average, 569 μg of filter OC was composited and extracted. Composites adequately represented each load demand cycle and fuel type and produced duplicates of each set of test conditions. All filter-based, organic compound emission factors are normalized to OC in this study. All OC and EC concentrations determined using thermal-optical analysis were artifact- and background-corrected. Artifact correction estimated the concentration of gas-phase OC adsorbed to the filter surface. It is not a total gas-phase OC measurement. Details of the background correction are provided in Section 2.6.

### 2.5. PM Extraction and GC–MS Analysis

The PM extraction and GC–MS conditions used for this investigation have been described exhaustively [4,13]. Briefly, each quartz filter composite was placed in a 50 mL glass jar and spiked with an internal standard mixture containing deuterated hydrocarbons, organic acids, and ^13^C-labeled anhydosugars such as levoglucosan. Internal standard spike volumes varied on the basis of anticipated final volume of extract. Use of the internal standard method allowed us to compensate for sample processing losses and changes in MS response over the analysis period. Filter composites (*n* = 3) were extracted twice (50 min and 5 min) ultrasonically with approximately 10 mL of a 2:2:1 vol/vol hexane, isopropanol, and benzene solution (HIB). Each extract was filtered with a 0.2 μm PTFE filter (Supelco, Iso-Disc™) and then concentrated to between 0.3 mL and 1 mL depending upon the OC concentration extracted. Each sample extract was split into two volumes. One volume underwent a series of derivatization reactions to convert the organic acids and anhydrosugars to their respective methyl ester and silyl-ester analogs. First, methylation was performed by reacting 50 μL of sample extract with 50 μL of in-house prepared diazomethane reagent and 15 μL of methanol, allowing the reaction to proceed for at least 1 h. Then, hydroxyl groups on levoglucosan were silyated by reacting 10 μL of methylated extract with 50 μL of BSTFA reagent (Sigma Aldrich, St. Louis, MO, USA). The reaction was allowed to proceed for 30 min at 70 °C, and then allowed to sit at room temperature overnight to ensure completion.

The neutral and both derivatized extracts were analyzed by GC–MS for a total of 115 organic compounds representing eleven compound classes. The compound classes included normal-alkanes, branched-alkanes, PAH, anhydrosugars, aromatic, resin, alkanoic, and fatty acids, aliphatic diacids, phytosterols, and methoxyphenols. The methoxyphenols were analyzed using thermal extraction (TE)-GC–MS (TDS3, Gerstel Inc., Baltimore, Maryland and Agilent Technologies ( Santa Clara, CA, United States) 6890/5973 MS [q]). For TE-GC–MS, a 1 μL volume of each sample extract was injected manually onto a baked Carbotrap F/Carbotrap C adsorbent tube. The solvent from each sample spike was evaporated by flowing nitrogen across each adsorbent tube for 60 s at a rate of 50 mL/min. All other organic compounds were analyzed using a GC–MS (Agilent 7673A/7000 series triple quadrupole [qqq]) system interfaced to a liquid sample autoinjector.

### 2.6. Quality Control and Study Caveats

Either an average response factor or linear regression was used for calibration and to quantify organic compound concentrations in the samples. The calibration range varied by target compound class. It was 0.1 to 1 ng μL for most PAH and 0.625 ng/μL to 6.25 ng/μL for most alkanes. A five-level levoglucosan standard range of 12.75 ng/μL to 130 ng/μL was used; the three-level organic acid calibration range was 2 ng/μL to 16 ng/μL. A mid-level continuing calibration of 10 ng/μL was used for methoxyphenol analytes. A mid-level check standard to assess target recovery was run daily. If the daily mid-level check standard failed to pass the minimum acceptance criterion (80% of compounds must agree to within 25% of actual fixed concentration value of standard), it was used as a daily continuing calibration that updated all target responses. Detection limits were determined for all target organic compounds as described in EPA document SW-846 with *N* = 7; *t* statistic = 3.14 [16]. Typical detection limits for the instrument used in this study were provided elsewhere [4]. Values below the detection limit threshold were reported as not detected (ND). Matrix spikes that considered all standard compounds were performed to determine extraction recovery. Matrix spike recoveries were used as an additional data quality check, and typical values were also reported previously [4]. Several of the methoxy phenols matrix spike targets were acceptable, whereas others were lower than expected.

Automated integration results for individual peaks were reviewed and corrected if applicable. Retention times were used for the identification of target analyte components. Because the GC was equipped with electronic programmable control (EPC), retention times shifted less than 0.1 min throughout the analysis period. Target analyte validity was also determined using fragment isotopic ratios that exceeded the minimum S/N ratio of 3:1 and had good proximity to mid-level check standard retention times. Additional quality control was performed by monitoring the internal standard response of all samples. Precision was demonstrated by triplicate injection checks of composite samples. Background correction was performed using dilution tunnel blank tests for all samples except for those burning HW pellets at full load and one test at low load. These emission factor values are given as is. In certain cases, background subtractions produced negative values. Negative values and non-detects were treated as “missing” during generation of descriptive statistics. Elution of individual phytosterol compounds was putatively observed for experiments conducted for both HW and SwG pellets. However, the vast majority of tests did not show these compounds, which were not reported here due to the lack of phytosterol standards.

This study measures individual SVOCs in the “filterable” fraction of the combustion emissions; thus, the SVOC term used here and throughout only refers to filter-based SVOCs. This fraction is often defined as “primary” or “condensable” PM and is of importance to the emissions policy and regulatory community. Gas-phase SVOCs were not included as part of these measurements. Note that several of the higher vapor pressure SVOCs under study presently were subject to equilibrium partitioning between the gas- and particle-phases as governed by thermodynamics. The partitioning behavior of these SVOCs is highly specific to the dilution, temperature, and other filter sampling and testing conditions. Thus, the SVOC concentrations in the filtered mass can vary substantially and are study-specific. Despite their high relevance to combustion equipment certification and conformity, the conditions used in this study are unlikely to represent atmospheric conditions. Certain gas-phase SVOCs can undergo atmospheric photoxidation, forming secondary organic aerosol (SOA). The potential for organic aerosol mass formation due to the gas-phase emissions is critical to understanding air quality issues but is not covered as part of this study.

### 2.7. Statistical Analysis

Statistical analyses were performed in JMP 14.0.0 (SAS Institute Inc., Cary, NC, USA). Concentration data were log-transformed, and test pairs of concentration means were compared using the Tukey–Kramer honest significant difference (HSD) test. The Tukey HSD test assumes the observations are independent and concentrations are normally distributed. The test is based on the studentized range distribution and used the critical *q* value to determine statistical significance at *α* = 0.05. Henceforth, the term “significant” is reserved for cases where the hypotheses were tested statistically.

## 3. Results

### 3.1. General BB Emissions Trends

Table 1 shows the mean BB emissions factors for PM, OC, and EC expressed in units of pollutant mass per mass of fuel burned. A description of the emission factor calculations is provided in Supplementary information. The calorific values of the pellets are provided in Table S1 so that a simple metric conversion can be applied if needed. The PM emissions ranged from 0.4 g/kg fuel to 2.91 g/kg fuel. The OC and EC emissions showed greater variation and accounted for 40% ± 17% *w/w* of the PM. Organic matter (OM) in the PM was estimated by multiplying OC by a factor of 1.8 [17], in which case the OM and EC accounted for 60% ± 31% *w/w* of the PM. Figure S1 provides the study-wide distributions, quantiles, and summary statistics for OC and EC concentrations in the BB emissions. Generally, OC levels were higher than EC levels during BB testing. The OC and EC concentrations varied from 3 to 2766 μg m^−3^ and from 8 to 1713 μg m^−3^, showing median concentrations of 550 μg m^−3^ and 90 μg m^−3^, respectively. The box plots in Figure S1 suggest the presence of outliers at the upper concentration limits for both OC and EC.

**Figure 1.**
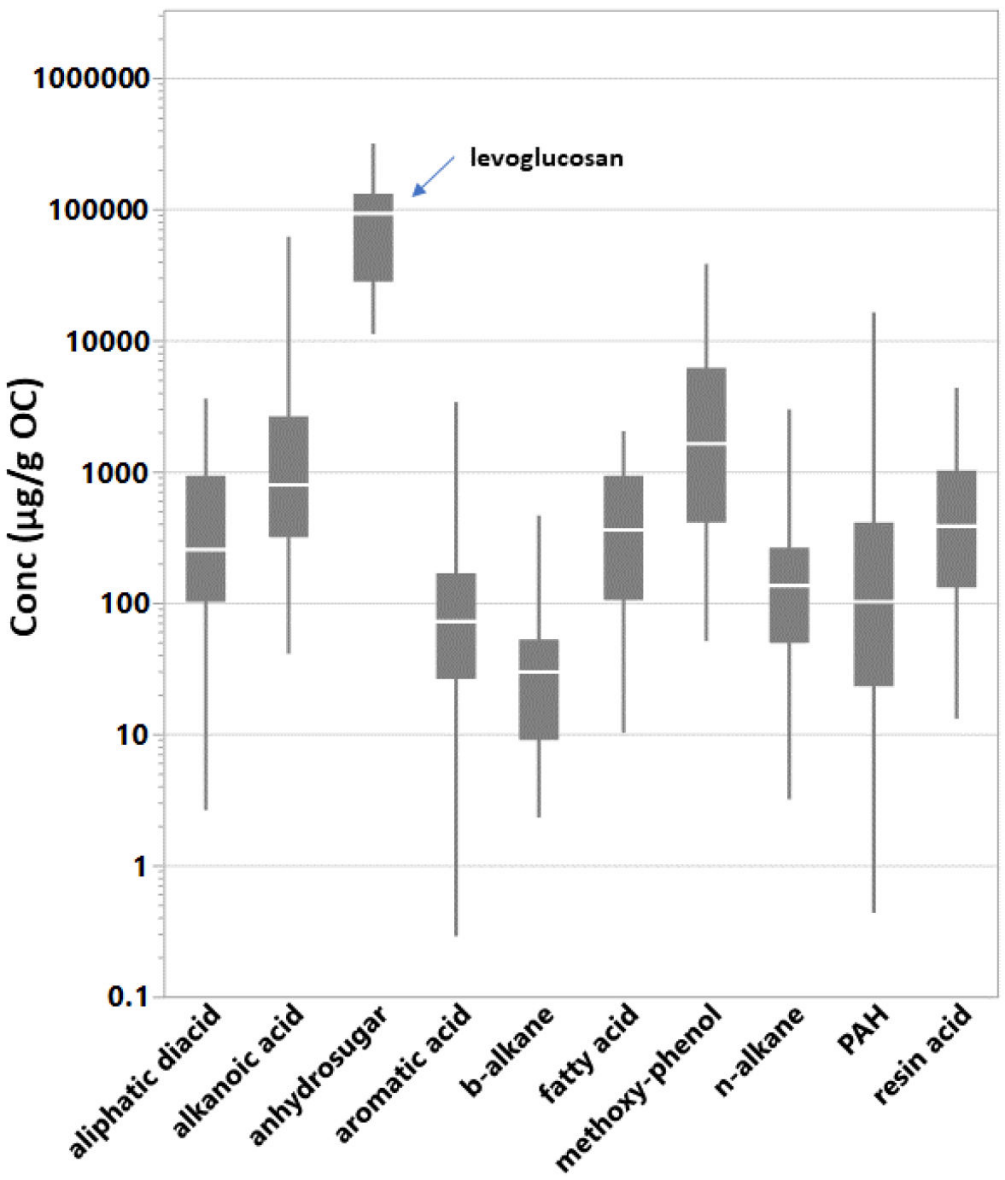
Quantile box plots of individual SVOC concentrations pooled by compound class. Levoglucosan is the anhydrosugar. The line in the box is at the median. The whiskers indicate the 10% and 90% quantiles. SVOC data populations (Table 2): aliphatic diacid (*n* = 81); alkanoic acid (*n* = 160); anhydrosugar (*n* = 15); aromatic acid (*n* = 103); *b*-alkane (*n* = 30); fatty acid (*n* = 48); methoxy-phenol (*n* = 84); *n*-alkane (*n* = 296); polycyclic aromatic hydrocarbons (PAH) (*n* = 332); resin acid (*n* = 38).

The sum of GC–MS-identified SVOCs (ΣSVOCs) in the filtered particles emitted from the BBs ranged from 0.13 g/g OC to 0.41 g/g OC (Table 1). Table 2 shows the population and range of individual SVOC concentrations by organic compound class. A total of 1187 individual compound concentrations were above detection limits and background levels. In general, oxygenated compounds were detected and quantified at higher concentrations than hydrocarbons; median concentrations were 436 μg/g OC and 108 μg/g OC, respectively. Table S2 provides the descriptive statistics for individual SVOC concentrations in the PM from BB testing, combining all experiments. Figure S2 presents mean concentrations (μg of compound/g OC) of the individual organic compounds in the BB fine PM emissions. Concentration ranges representing all test conditions are indicated by the whiskers and varied by greater than 3 orders of magnitude for nearly half of the compounds. The vast majority of compound concentration means were within 10 and 1000 μg/g OC. The concentration of levoglucosan was highest and at least 5 times greater than any other individual SVOC concentration. Levoglucosan is the only compound representing the anhydrosugars. Individual SVOC emission levels within the alkanoic acids, methoxy-phenols, and PAH classes followed levoglucosan in that order. The ΣSVOCs class emissions (Table 2) varied by as much of 2 orders of magnitude during testing. Figure 1 pools the individual compound concentrations by compound class and shows the relative enrichment of the methoxy-phenols and organic acids.

### 3.2. Effect of Pellet Fuel Type on Emissions

**Figure 2.**
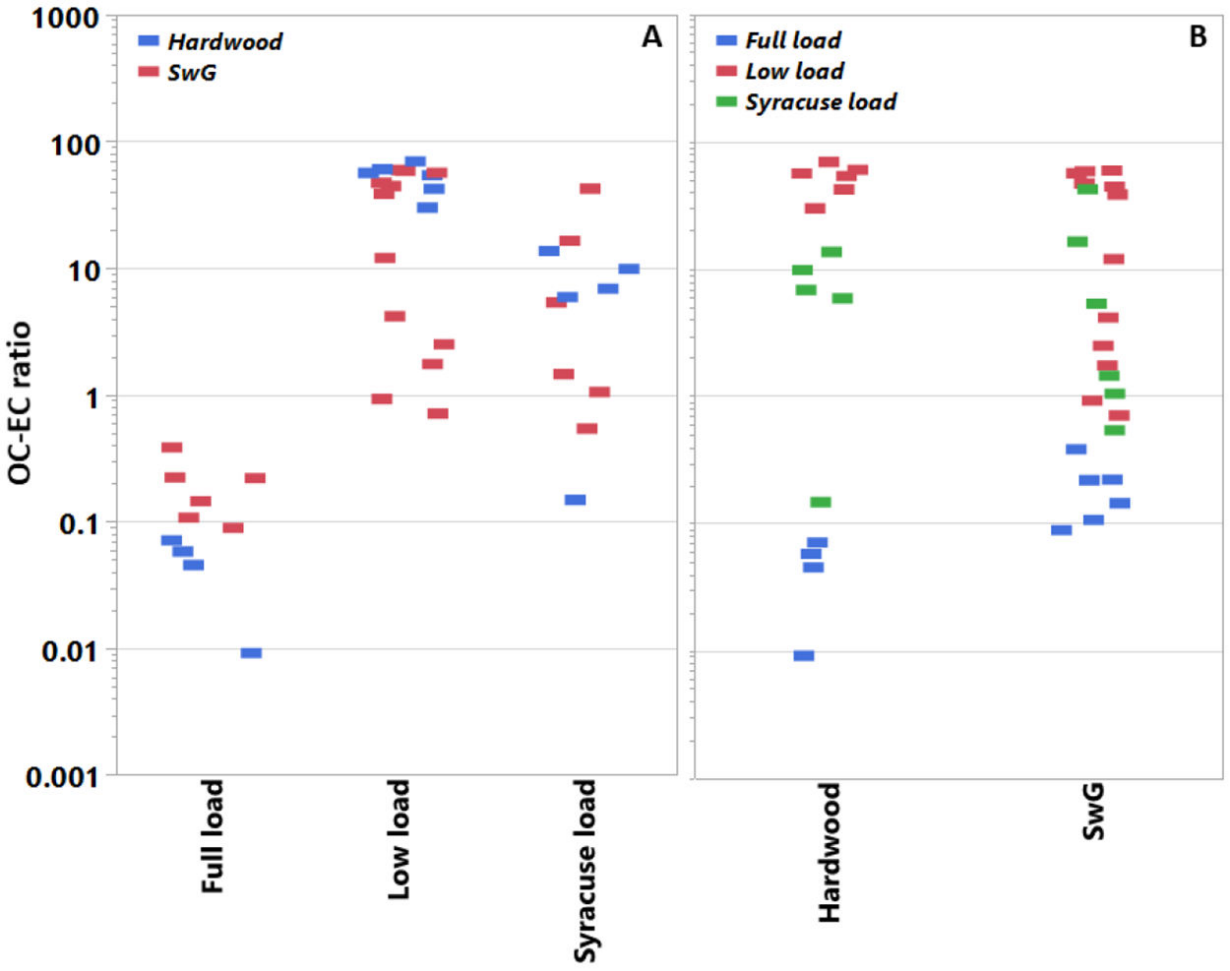
Filter-based OC–EC ratios in PM for individual tests sorted by heat load demand profile and fuel type. Panel A pools the OC–EC ratios by fuel type, whereas panel B pools them by operational mode. Data populations: Full load (*n* = 10), low load (*n* = 18), and Syracuse load (*n* = 11).

On average, the fuel type showed minimal effect on the PM mass emissions (Table 1). Figure 2 shows the OC–EC ratios for each pellet fuel type at each load condition and vice versa. The OC–EC ratio was sensitive to the use of the different pellets as the load conditions varied. This point will be discussed further below with the test cycle effects. With the load conditions pooled, pellet type showed no significant influence on the OC–EC ratio.

**Figure 3.**
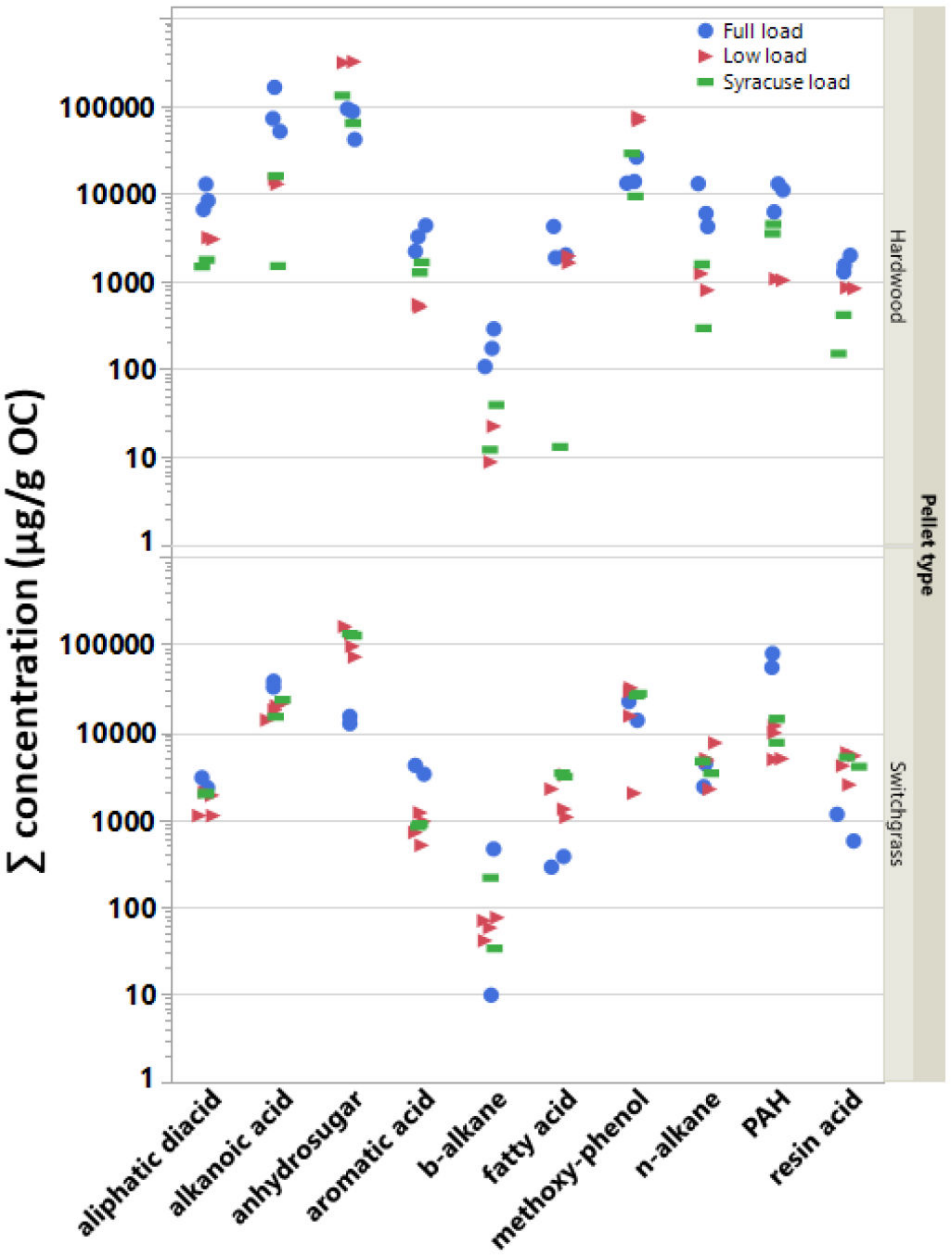
Concentration sums (μg/gOC) for individual tests sorted by compound class, test load conditions, and fuel type (HW: hardwood pellet; SwG: switchgrass pellet).

Like the OC–EC ratio, there is no effect on the measured SVOC emissions due to pellet type if all SVOC concentrations and test cycles are combined. Thus, individual SVOC compounds were summed, averaged, or pooled within their respective classes in order to further evaluate the effects of pellet type (and load conditions) on the test emissions. Figure 3 shows the ΣSVOC emissions for each BB test by compound class, test load condition, and pellet fuel type. The Tukey HSD test results confirmed that the effect of pellet fuel on the emissions varied by compound class. Specifically, it indicated (i) significantly higher aliphatic diacid, alkanoic acid, and methoxy phenol mean concentrations in the HW pellet emissions; (ii) significantly reduced PAH concentrations in the BB emissions due to burning HW pellets; and (iii) no effect due to pellet type for levoglucosan, aromatic, resin and fatty acids and *b*- and *n*-alkanes concentrations in the aerosol emissions. Several of these observations can also be visualized in Figure 3.

### 3.3. Effect of Test Cycle on Emissions

For both fuels, low load conditions produced higher PM emissions (Table 1). Test cycle also influenced the OC–EC emissions trends as indicated by Figure 2. Tests at low load (25%) produced significantly more OC in the PM emissions than either the Syracuse or full loads tests for the BB, averaging across fuel type; the full load cycle (100%) produced more EC than OC. Under both full- and low-load conditions, the effect of pellet type on the OC–EC ratios was significant. At full load, the HW pellets generated relatively less OC and more EC than the SwG pellets. At low load, the trend is reversed; that is, at low load the HW pellets produce more OC and less EC than the SwG fuel (e.g., see Table 3).

The mean concentrations of resin acids, fatty acids, and methoxy-phenols in the organic aerosol particle emissions showed no significant difference under the different BB test load conditions used in the present study. However, the full load conditions produced significantly higher mean concentrations (μg/g OC) than both Syracuse and low load conditions for several compound classes (aliphatic diacids, alkanoic acids, *n*-alkanes, and PAH). Moreover, compared with full load testing, low load tests produced significantly higher levoglucosan and lower aromatic acid concentrations in the organic PM. Finally, a one-way analysis using a data pool including all measured organic compounds irrespective of class showed no significant difference among pairs of means representing test load conditions and pellet type.

## 4. Discussion

High efficiency, lower emissions, and ease of use due to automation of fuel and air delivery are among the benefits afforded by pellet burning appliances [4]. Yet, studies evaluating the energy and biomass burning emissions performance of BBs are relatively scant, and even fewer studies examine the composition of organic species in BB emissions using alternative fuels like non-woody biomass pellets. Alternative fuels are often perceived as carbon-neutral and are growing in popularity, thus warranting further investigation.

In the present study, the BB unit performance is optimized at full load, explaining why the PM emissions increase at lower load (Table 1). Orasche et al. [11] also show that operation at nominal load tends to reduce PM emissions. PM emissions factors (g/kg) from multiple (*n* = 8) studies that tested a variety of pellet and biomass boilers and residential wood burning appliances are presented in Figure S3. The figure shows that PM emissions from the different biomass burning appliances used across studies vary over at least 2 orders of magnitude. Generally, pellet-burning significantly reduces PM emissions (0.1–5.2 g/kg) compared with wood combustion in wood-stoves, fireplaces, or boilers (0.2–47 g/kg). The mean PM mass emissions value produced using the current BB (1.1 g/kg) is well within the range produced for pellet burning studies despite the wide variety of pellet types burned, including wood, grass, corn stalk, sunflower stalk, sewage sludge, and straw. Consequently, the pellet fuel type does not appear to be a critical variable governing PM emissions. Compared with distillate oil boilers, which are being replaced with alternatives in some U.S. states, the PM emissions from the pellet-fired BB are an order of magnitude greater on average [18].

Compared with the reports of PM mass emissions, relatively fewer studies investigate the OC and EC composition of the PM emitted from biomass boilers. Those studies available show, on average (see Figure S4, panel A), that boilers emit PM comprising less total carbon compared with PM from residential wood stoves or fireplaces [19]. For example, this study and Orasche et al. [11] report ranges of 2–75% *w/w* and 16–63% *w/w*, respectively, for total carbon in boiler PM; whereas, the total carbon in PM emitted during a typical residential wood burning study is given as 91–113% *w/w* [19]. As expected, controlled combustion in a boiler or furnace is generally more efficient than in a residential appliance. Although, the OC–EC ratios observed (Figure S4, panel B) for the current study and for past boiler emissions work are > 1, indicating that smoldering fire conditions also occur in boilers. The similar carbon content in the HW (48%) and SwG (43%) pellet fuels used here may explain the lack of influence that fuel type had on the mean OC–EC ratio for composite test conditions. As mentioned, the BB unit tested presently is optimized to operate at full load; thus, it emits less PM at full load, and that the PM emitted comprises higher EC levels because much of the semivolatile organic matter is presumably oxidized (see Figure 2). Higher levels of OC in PM are produced as the BB efficiency decreases with the lower heat demand profile. The carbon-based molecular species in each fuel type likely differed as HW pellets show higher OC at low load, but lower OC at high load conditions compared with the SwG pellets, see Table 3. Perhaps the carbon composition of the SwG fuel is less susceptible to thermal degradation. For example, the balance of natural binding components in pellet fuels commonly vary. Compared with wood, SwG pellets tend to produce more ash that is silicon-based and has lower fusion temperatures (see Table S1) [8]. Thus, slagging while burning SwG may also reduce the heating rate and combustion efficiency in the BB.

The SVOC emissions examined here are byproducts of incomplete combustion. The concentrations and distribution of SVOC byproducts are determined by temperature, heating rate, and stoichiometry among other factors [20]. As for the enrichment of SVOCs in the PM organic carbon, the PAH and organic acid compound groups appear somewhat sensitive to changes in test cycle conditions and pellet fuel. At high load conditions, higher combustion zone temperatures are produced that favor the formation and growth of PAH and soot nuclei. Aromatic PAH compounds have high thermal stability due to their resonance structure. Hence, it is possible that comparable levels of PAH are forming across load conditions, but compounds with lower dissociation energy bonds (i.e., C-C bonds) are thermally degrading at the higher loads, thereby reducing OC mass [21]. The relative reduction in PAH observed with the HW pellet use is likely due to an increase in competing oxidation processes or corresponding decrease in the concentration of low molecular weight (C_2_; C_3_) gaseous PAH precursors [22]. Additionally, the relatively high concentration of lignin and thus aromatics in the HW pellets may offer a pathway to producing soot with heavier PAH (e.g., with molecular weight > 500 amu), which are not measured with the GC–MS methodology applied currently. The higher lignin concentrations in the HW pellets may also explain the generally higher concentrations of aromatic acid and methoxyphenol subunits in HW pellet burning emissions, which indicate the thermal breakdown of the lignin polymer structure. The *n*-alkanoic acids originate from plant fats, oils, and phospholipids and are prevalent in biomass burning emissions; additionally, the even-to-odd C number predominance and C_*max*_ = C_16_ observed here is a commonly observed feature of the *n*-alkanoic acids grouping [23].

**Figure 4.**
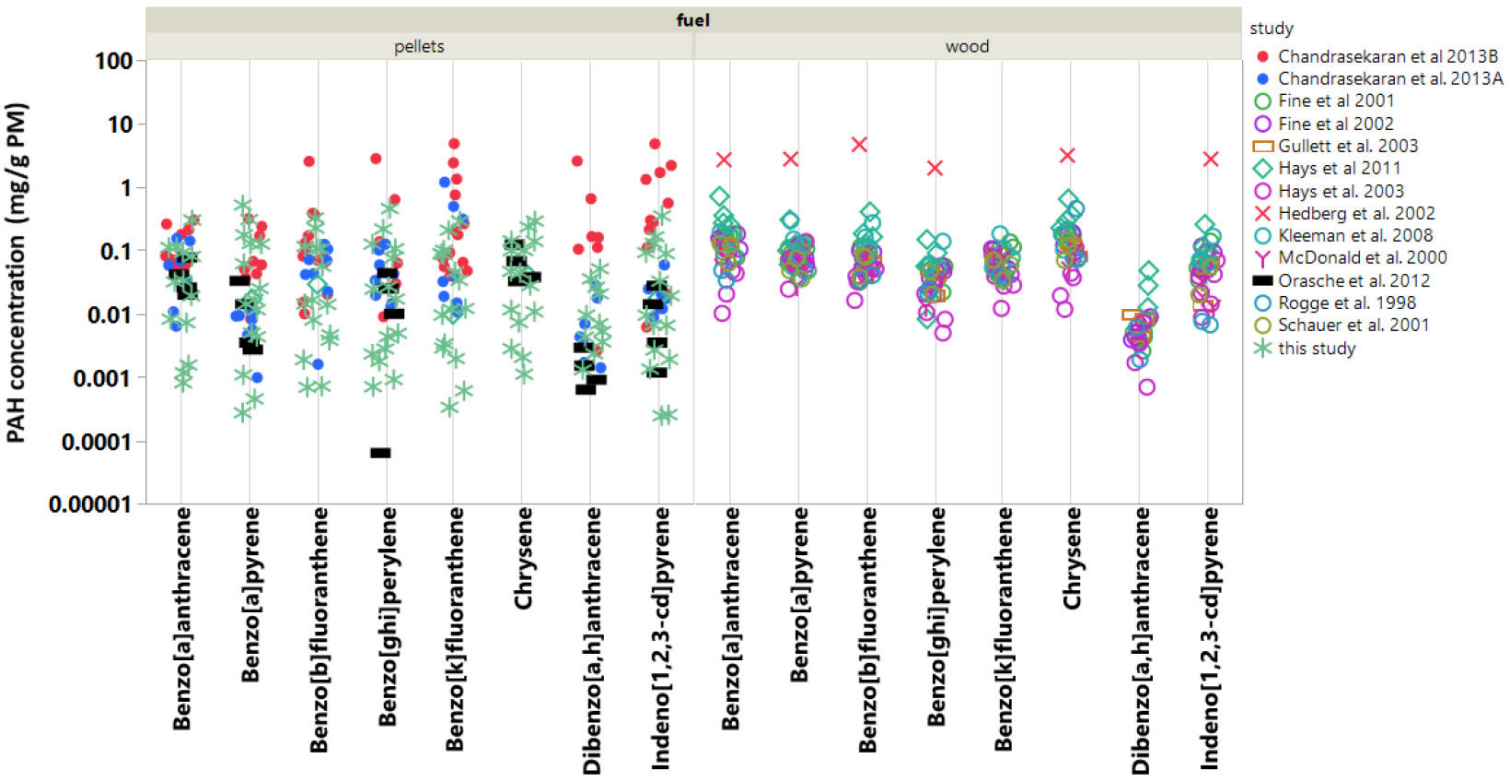
Comparison of PAH concentrations in PM (mg/g PM) emitted from wood- and pellet-burning appliances. Data populations for individual compounds ranged from *n* = 57 to *n* = 92. Figure S5 shows the calculated means with standard error and median values for individual PAH concentrations gathered across the multiple studies. These values are provided in an effort to highlight differences and provide consensus.

Of the thousands of chemical constituents in PM, few have received more attention than the PAH compounds due to their carcinogenicity and strong contribution to mutagenicity [24]. In fact, earlier work shows that residential wood burning produces particle emissions that are among the most mutagenic (reversants/milliJoule) [25]. To address the toxicological concern due to PAH in biomass burning particle emissions, eight select PAH emitted from pellet burning appliances [9-11] are compared to those emitted from residential wood burning in fireplaces, woodstoves, and hydronic heaters [4,19,26-33] (Figure 4). The PAH compounds selected for further comparison are designated EPA priority PAH with relatively low vapor pressures (< 2.5 × 10^−6^ mm Hg @ 25 °C), which ensures that these compounds predominate in the nonvolatile particle fraction. Figure 4 shows several interesting features. First, the individual PAH in PM emitted from pellet burning varied over a substantially wider range than the PAH from wood burning. This indicates the variety of operating conditions and boiler types (*N* = 11) used across the pellet burning studies. The pellet burning also produced on average 2–10 times more benzo[*k*]fluoranthene, dibenz[*a,h*]anthracene, and indeno[*1,2,3-c,d*]pyrene in PM. All three compounds are carcinogenic, and dibenzo[*a,h*]anthracene is estimated as 10 times more toxic than benzo[*a*]pyrene [34]. Note that, for each fuel type, the median values for these three compounds are lower than the means and at least a factor of two lower for the pellet burning studies (see Figure S5). The calorific values of the fuels in the comparison were narrowly distributed (14–19 MJ/kg) with systematically higher values for pellet fuels. Thus, wood burning studies would produce marginally higher emissions per MJ of fuel. Benzo[*a*]pyrene is a factor of three lower in the PM from pellet burns. Pellet burning in BBs also produces some of the lowest PAH levels in PM compared with indoor residential wood burning and wood burning in hydronic heaters [4]. This is likely due to the relative efficiency of burning pellets. Moreover, the wood burning hydronic heaters tested had higher nominal output ratings, were manually charged as opposed to automatically fed, and sometimes utilized longer Syracuse duty cycles (12 h and 24 h), all of which potentially contributed to higher PAH emissions. Despite the lower PM mass emissions typical of pellet burning, these results show that the PAH distribution in the PM from pellet burning differs substantially from wood burning in BBs and residential wood stove and fireplace appliances, and thus should undergo further toxicological evaluation. Finally, among the pellet-burning studies, Chandrasekaran et al. [10] report the highest PAH concentrations in PM (up to 10 mg/g of PM). A single 30 kW boiler produced these results while operating at high load with fixed, continuous air flow; the boiler was also considered efficient as it achieved a notably high operating temperature of 1100 °C. On average, the PAH results of the present study agree remarkably well with Orasche et al. [11] and Chandrasekaran et al. [9]. Although, for six of the eight PAH compounds examined, our new measurements are among the lowest PAH concentrations in PM emissions from BBs.

## 5. Summary

Pellet burning in the BB generated fine PM mass that contained less carbon than traditional domestic biomass burning appliances. Despite fuel composition differences, the pellet type used in the BB had less influence on the emissions than the test cycle variable. For example, EC—a short-term climate forcing agent—was emitted in relatively high quantities at nominal loads; whereas, at low load conditions, the BB produced more fine PM that contained significantly higher OC. The majority of GC–MS identified SVOCs emitted from the BB during pellet burning were oxygenated compounds, including organic acids, methoxyphenols, and levoglucosan. The evidence shows that PAH in PM from pellet-burning varies over a wide range compared with what is typically observed for residential biomass burning. Eight PAH were selected for further analysis owing to their low volatility and known toxicity. Compared with other forms of biomass burning, pellet burning in the BB generated lower levels of benzo[*a*]pyrene but higher levels of benzo[*k*]fluoranthene, indeno[*1,2,3-c,d*]pyrene, and dibenz[*a,h*]anthracene. Because biomass-burning particles are among the most mutagenic, and the distribution of PAH in BB emissions tends to differ from those from other residential biomass burning, further toxicological investigation is likely needed.

## Supplementary Material

Sup1

## Figures and Tables

**Table 1.** Mean BB emission factors for PM, OC, and EC given as a function of fuel type and load cycle. Error is expressed as a relative standard deviation (RSD). The ΣSVOCs identified and quantified by GC–MS are also provided. Full load testing with HW pellets were run in triplicate, and *n* = 4 for low load testing with Switchgrass pellets. *n* = 2 for all other tests. Data from the analysis of multiple filters was used to determine the mean and standard deviations. SyrC = Syracuse cycle.

Load	Fuel	PM		OC		EC		ΣSVOCs	
		(g/kg Fuel)		(mg/kg Fuel)		(mg/kg Fuel)		(g/g OC)	
25%	Hard wood	2.91	±2%	1075	±46%	20	±45%	0.41	±3%
SyrC		0.269	±25%	33.1	±122%	10.2	±93%	0.13	±38%
100%		0.401	±11%	1.78	±200%	90	±96%	0.22	±43%
25%	Switch grass	1.3	±35%	572	±20%	11.1	±24%	0.17	±30%
SyrC		0.761	±24%	392	±38%	83.8	±84%	0.20	±5%
100%		0.662	±40%	62.8	±115%	292	±45%	0.15	±1%

**Table 2.** The range of individual semivolatile organic compound (SVOC) concentration observations and test-based sum of SVOC concentrations (*N* = 15) by compound class. Note that the anhydrosugar class contains levoglucosan only.

Compound Class	Individual SVOC concs.			ΣSVOC Class		
	*n*	Min	Max	Min	Max	Median
	(μg/g OC)					
aliphatic diacid	81	3	3638	1106	12,849	2056
alkanoic acid	160	41	61,161	1494	163,709	19,878
anhydrosugar	15	12,505	320,300	-	-	95405
aromatic acid	103	0.291	3417	508	4360	1197
*b*-alkane	30	2	461	9	461	58
fatty acid	48	10	2019	13	4238	1906
methoxy-phenol	84	52	38,282	1998	74,902	26,056
*n*-alkane	296	3	2962	294	13,044	4231
PAH	332	0.4	16,590	1036	79,021	7589
resin acid	38	13	4303	150	5783	1526

**Table 3.** Mean, standard deviation, and range of OC and EC concentrations in emissions from the BB testing.

Fuel	Load	*n*	OC	EC
			(μg m^−3^)	
Hardwood	25%	6	1478 ± 773	27.6 ± 10.6
	Syracuse	6	69 ± 36	20 ± 24
	100%	9	11.3 ± 14.3	244.6 ± 220.1
Switch grass	25%	12	758 ± 350	138 ± 176
	Syracuse	6	996 ± 984	212 ± 200
	100%	6	297 ± 209	142 ± 325
