# Supplementary material for: Carbonaceous Particulate Matter Emitted from a Pellet-Fired Biomass Boiler
Source: Atmosphere (Basel). Author manuscript; Available in PMC 2021 Feb 23. (PMC7900939; doi:10.3390/atmos10090536)
Supplement: Sup1 [file NIHMS1546983-supplement-Sup1.pdf]

# Supplementary Materials:

# Carbonaceous particulate matter emitted from a pellet-fired biomass boiler

**Michael D. Hays[1,*], John Kinsey[1], Ingrid George[1], William Preston[2], Carl Singer[2] and Bakul Patel[3]**

Contents

Schematic 1 2

Figure S1 **Error! Bookmark not defined.**

Figure S2 **Error! Bookmark not defined.**

Figure S3 **Error! Bookmark not defined.**

Figure S4 **Error! Bookmark not defined.**

Figure S5 **Error! Bookmark not defined.**

Table S1 **Error! Bookmark not defined.**

Table S2 **Error! Bookmark not defined.**

Emission factor calculations 11

Dilution Factor 11

References 12

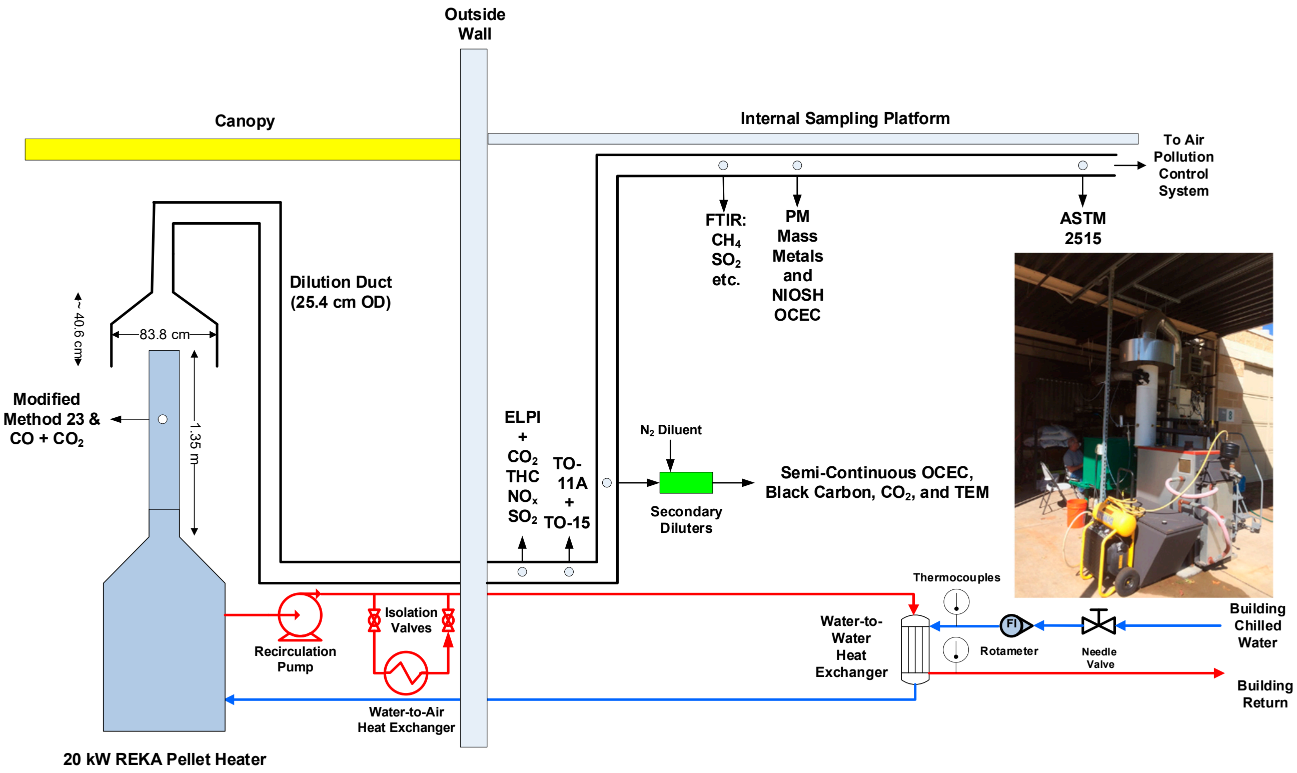


**Schematic 1**. Pellet boiler testing facility and BB photo.

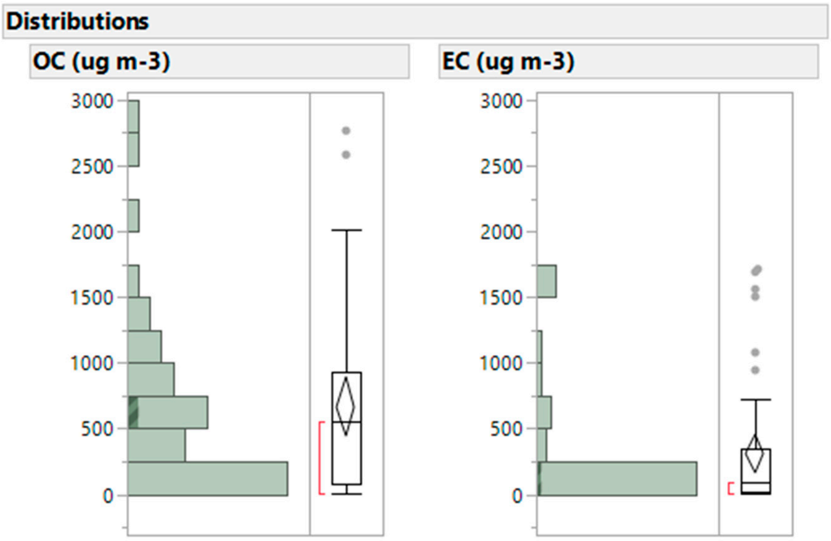


**OC (ug m-3) — Quantiles**

| | | |
|---|---|---|
| 100.0% | maximum | 2765.88 |
| 99.5% | | 2765.88 |
| 97.5% | | 2765.88 |
| 90.0% | | 1727.46 |
| 75.0% | quartile | 928.84 |
| 50.0% | median | 550.18 |
| 25.0% | quartile | 85.89 |
| 10.0% | | 10.25 |
| 2.5% | | 2.99 |
| 0.5% | | 2.99 |
| 0.0% | minimum | 2.99 |

**OC (ug m-3) — Summary Statistics**

| | |
|---|---|
| Mean | 669.45359 |
| Std Dev | 696.22215 |
| Std Err Mean | 111.48477 |
| Upper 95% Mean | 895.1427 |
| Lower 95% Mean | 443.76448 |
| N | 39 |

**EC (ug m-3) — Quantiles**

| | | |
|---|---|---|
| 100.0% | maximum | 1713.28 |
| 99.5% | | 1713.28 |
| 97.5% | | 1710.0565 |
| 90.0% | | 1249.18 |
| 75.0% | quartile | 350.66 |
| 50.0% | median | 89.56 |
| 25.0% | quartile | 20.92 |
| 10.0% | | 11.98 |
| 2.5% | | 7.927 |
| 0.5% | | 7.78 |
| 0.0% | minimum | 7.78 |

**EC (ug m-3) — Summary Statistics**

| | |
|---|---|
| Mean | 309.042 |
| Std Dev | 482.13915 |
| Std Err Mean | 71.87306 |
| Upper 95% Mean | 453.89264 |
| Lower 95% Mean | 164.19136 |
| N | 45 |

**Figure 1.** Study-wide distributions, quantiles, and summary statistics for OC-EC concentrations in the PHBB emissions. In the outlier box plot, the confidence diamond contains the mean and the 95% confidence interval about the mean. The red bracket outside the box is referred to as the 'shortest half' or the densest 50% of the observations.

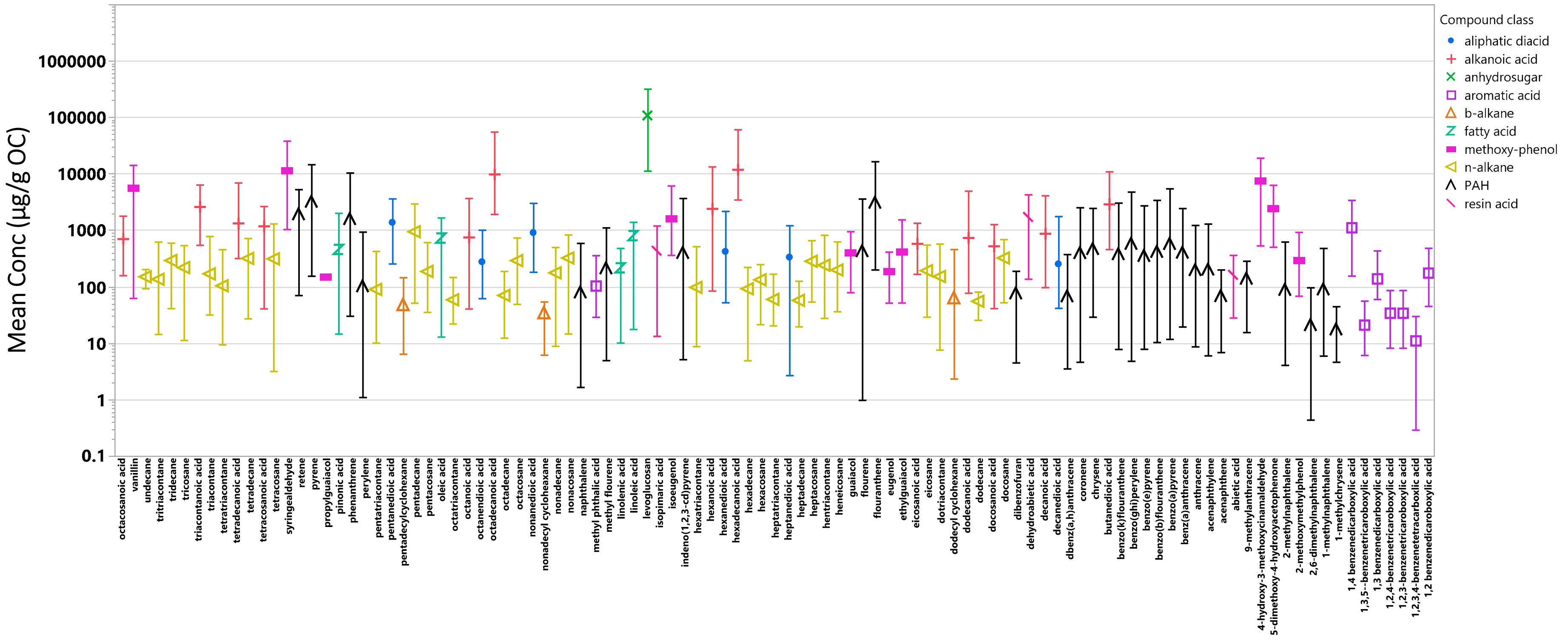


**Figure 2.** Individual mean SVOC concentrations in PM emitted from boiler testing. Concentrations are given in units of g/g OC. Whiskers indicate the concentration range. The *y*-axis is log scale. Symbols and colors are coded by compound class. The descriptive statistics for each individual compound is provided in Table S2.

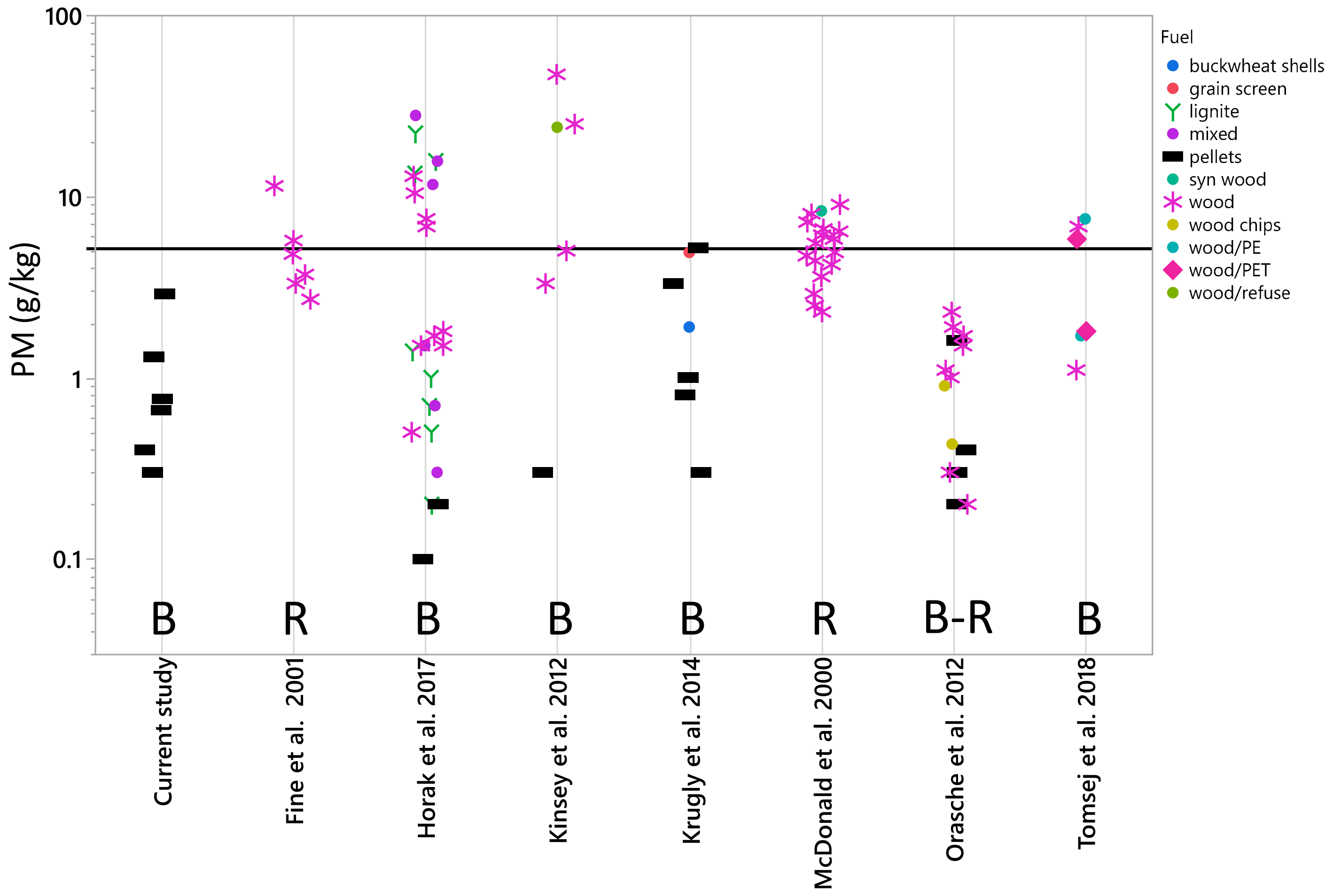


**Figure 3.** PM emissions factors (g/kg) for a variety of biomass burning studies (*n*= 87) examining residential (R) fireplace and woodstove and commercial and residential boiler (B) appliances. Fuel type is coded by symbol type and color. Fine et al. 2001[1] and McDonald et al. 2000[2] investigated residential fireplaces and woodstoves. Kinsey et al. 2012[3] tested outdoor wood hydronic heaters (38 kW-50 kW). Tomsej et al. 2018[4] examined a 20 kW commercial boiler. Horak et al. 2017[5] looked at a variety of 20 kW boiler types, including a gasifier, and auto-, over-fire, and down-draft boilers. Krugly et al. 2014[6] looked at a 13 kW boiler, and Orasche et al. 2012[7] studied a boiler and residential stove. The focus is only on studies that report EFs in units of w/w. Horak et al. 2017[5] boiler power is estimated based on nominal power output. Orashe et al. 2012[7] values are converted using their provided fuel calorific value. All values are presented as averages and represent a wide variety of power outputs. The solid line at 5.2 g/kg is the study-wide mean; the median was 2.5 g/kg. .

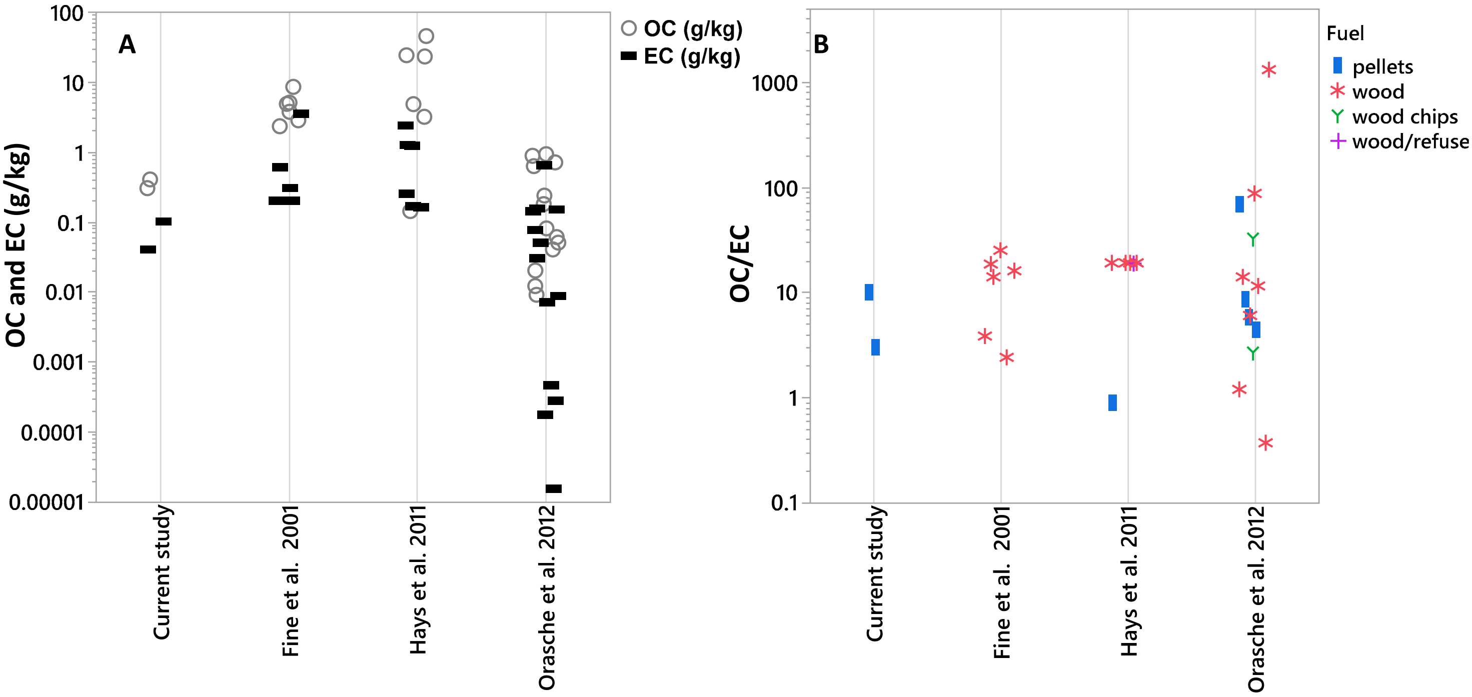


**Figure 4.** Literature comparison of organic and elemental carbon emission factors (g/kg, Panel A) and OC/EC emissions ratios (Panel B) for PM emitted from biomass burning in residential fireplaces and boiler appliances. Values for the current study are the studies in Figure 3 means by pellet type. In panel B, fuel type is coded by symbol type and color.

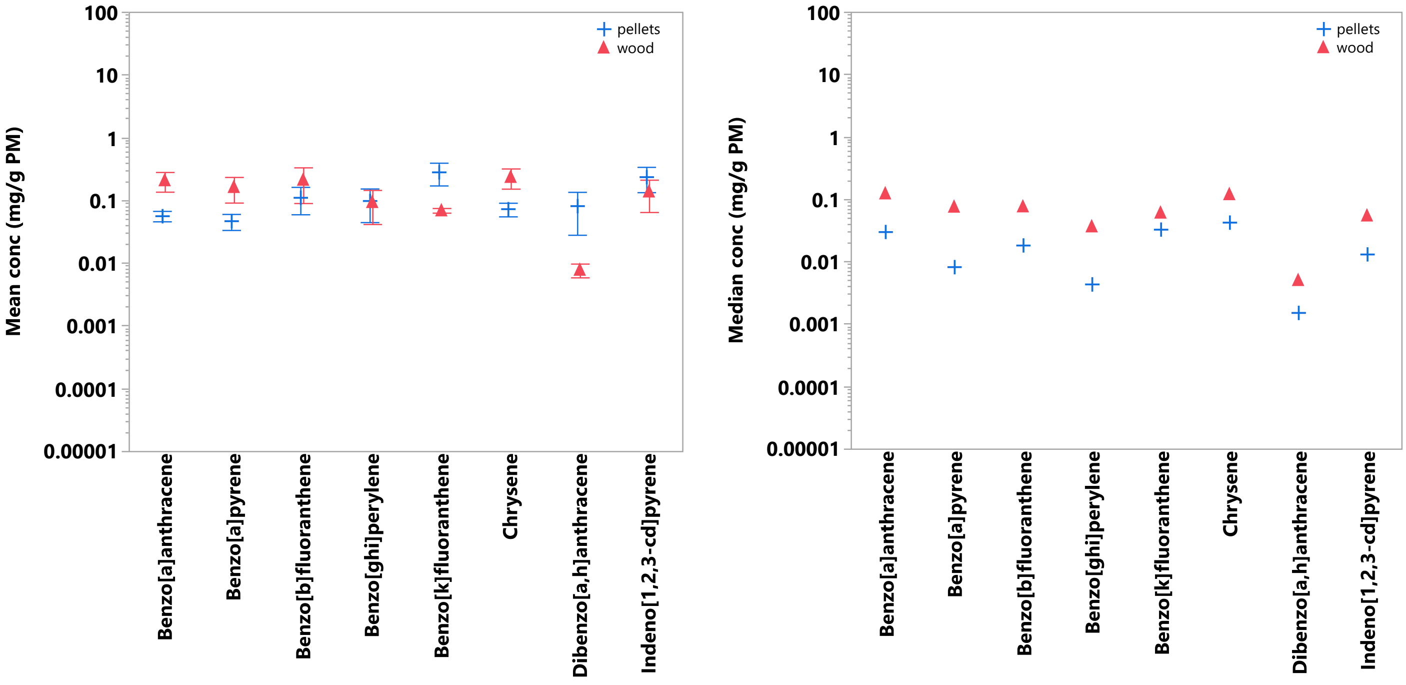


**Figure 5.** Calculated mean (with standard error) and median values of individual PAH concentrations as determined using the literature values provided in Figure 4. Values are grouped by fuel type.

**Table 1.** Fuel Analysis Results.

| Parameter Measured | Hardwood Pellets | Switchgrass Pellets |
|---|---|---|
| Proximate Analysis (as received) | | |
| Moisture | 4.66% | 10.41% |
| Volatile Matter | 80.34% | 72.62% |
| Fixed Carbon | 14.00% | 12.71% |
| Ash | 1.00% | 4.26% |
| Ultimate Analysis (as received) | | |
| Sulfur | 0.09% | 0.12% |
| Carbon | 47.91% | 42.79% |
| Hydrogen | 5.79% | 5.44% |
| Nitrogen | 0.31% | 0.82% |
| Oxygen (by difference) | 40.24% | 36.16% |
| Gross Calorific Value (MJ/kg) | 17.99 | 17.51 |
| Ash Mineral Analysis | | |
| Silicon Dioxide | 26.57% | 62.99% |
| Aluminum Oxide | 2.40% | 7.55% |
| Ferric Oxide | 4.89% | 3.63% |
| Titanium Dioxide | 0.27% | 0.06% |
| Phosphorus Pentoxide | 0.80% | 1.00% |
| Calcium Oxide | 36.63% | 10.69% |
| Magnesium Oxide | 2.58% | 3.65% |
| Sodium Oxide | 0.44% | 1.41% |
| Potassium Oxide | 11.76% | 6.42% |
| Sulfur Trioxide | 13.91% | 2.32% |

**Table 2.** Descriptive statistics for individual SVOC concentrations in the PM emitted from boiler testing. All experiments are combined. Concentrations are given in units of g/g OC.

| Compound | N | Mean | Minimum | Maximum | Median |
|---|---|---|---|---|---|
| | | | Concentration (µg/g OC) | | |
| 1,2 benzenedicaroboxylic acid | 15 | 175.27 | 45.10 | 485.51 | 111.03 |
| 1,2,3,4-benzenetetracarboxlic acid | 4 | 11.09 | 0.29 | 30.03 | 7.02 |
| 1,2,3-benzenetricaroboxylic acid | 13 | 37.76 | 8.22 | 86.51 | 37.58 |
| 1,2,4-benzenetricaroboxylic acid | 13 | 37.92 | 8.22 | 86.51 | 38.22 |
| 1,3 benzenedicarboxylic acid | 13 | 145.44 | 60.02 | 436.48 | 101.69 |
| 1,3,5--benzenetricaroboxylic acid | 15 | 23.14 | 6.50 | 56.04 | 15.48 |
| 1,4 benzenedicarboxylic acid | 15 | 1249.44 | 155.62 | 3417.97 | 957.36 |
| 1-methylchrysene | 14 | 18.68 | 4.64 | 44.63 | 18.53 |
| 1-methylnaphthalene | 8 | 98.49 | 5.96 | 480.86 | 51.27 |
| 2,6-dimethylnaphthalene | 14 | 20.62 | 0.44 | 96.47 | 7.86 |
| 2-methoxymethylphenol | 6 | 295.80 | 68.61 | 929.65 | 193.52 |
| 2-methylnaphthalene | 11 | 94.53 | 4.08 | 626.51 | 24.78 |
| 3,5-dimethoxy-4-hydroxyacetophenone | 9 | 2426.72 | 504.81 | 6341.00 | 1687.61 |
| 4-hydroxy-3-methoxycinamaldehyde | 12 | 8472.37 | 536.46 | 19161.90 | 7141.53 |
| 9-methylanthracene | 3 | 137.56 | 15.58 | 286.19 | 110.92 |
| abietic acid | 11 | 165.33 | 28.13 | 362.59 | 145.11 |
| acenaphthene | 4 | 67.54 | 6.87 | 200.32 | 31.48 |
| acenaphthylene | 9 | 198.74 | 6.01 | 1298.41 | 18.64 |
| anthracene | 14 | 217.84 | 8.70 | 1231.92 | 52.00 |

| | | | | | |
|---|---|---|---|---|---|
| benz(a)anthracene | 14 | 415.69 | 19.55 | 2450.37 | 218.71 |
| benzo(a)pyrene | 15 | 628.62 | 11.79 | 5478.19 | 119.49 |
| benzo(b)flouranthene | 15 | 446.47 | 10.35 | 3428.09 | 155.81 |
| benzo(e)pyrene | 15 | 367.59 | 7.85 | 2736.06 | 151.04 |
| benzo(ghi)perylene | 15 | 611.26 | 4.82 | 4808.73 | 171.32 |
| benzo(k)flouranthene | 15 | 406.68 | 7.81 | 3059.67 | 137.92 |
| butanedioic acid | 14 | 3163.55 | 461.53 | 10961.41 | 2316.96 |
| chrysene | 15 | 454.21 | 29.17 | 2468.34 | 251.03 |
| coronene | 14 | 410.16 | 4.65 | 2529.31 | 134.61 |
| dbenz(a,h)anthracene | 11 | 66.80 | 3.54 | 375.23 | 29.31 |
| decanedioic acid | 14 | 273.01 | 41.76 | 1766.97 | 99.18 |
| decanoic acid | 13 | 969.47 | 97.02 | 4135.51 | 450.11 |
| dehydroabietic acid | 15 | 1863.13 | 136.90 | 4303.41 | 1525.64 |
| dibenzofuran | 6 | 75.42 | 4.51 | 189.05 | 50.62 |
| docosane | 14 | 323.74 | 52.61 | 690.26 | 289.11 |
| docosanoic acid | 13 | 509.91 | 41.43 | 1273.86 | 417.62 |
| dodecane | 3 | 54.54 | 25.66 | 81.19 | 56.76 |
| dodecanoic acid | 13 | 827.99 | 77.59 | 4988.45 | 267.92 |
| dodecyl cyclohexane | 15 | 65.36 | 2.34 | 461.21 | 12.07 |
| dotriacontane | 13 | 150.22 | 7.61 | 575.14 | 87.70 |
| eicosane | 14 | 186.74 | 29.15 | 554.46 | 174.58 |
| eicosanoic acid | 14 | 631.12 | 167.77 | 1343.29 | 411.58 |
| ethylguaiacol | 6 | 420.25 | 51.98 | 1543.88 | 243.72 |
| eugenol | 4 | 186.40 | 51.55 | 415.02 | 139.51 |
| flouranthene | 15 | 3086.69 | 200.58 | 16589.76 | 1409.59 |
| flourene | 11 | 421.14 | 0.98 | 3618.80 | 13.05 |
| guaiacol | 7 | 302.15 | 78.99 | 796.84 | 257.73 |
| heneicosane | 15 | 195.57 | 36.34 | 627.50 | 189.68 |
| hentriacontane | 14 | 237.92 | 27.76 | 823.83 | 120.24 |
| heptacosane | 10 | 278.93 | 54.06 | 660.25 | 266.45 |
| heptadecane | 12 | 56.85 | 19.65 | 126.56 | 39.85 |
| heptanedioic acid | 10 | 379.41 | 2.69 | 1219.78 | 135.31 |
| heptatriacontane | 5 | 59.14 | 20.39 | 168.23 | 34.78 |
| hexacosane | 10 | 131.19 | 21.43 | 248.70 | 123.07 |
| hexadecane | 14 | 81.63 | 4.92 | 222.79 | 45.38 |
| hexadecanoic acid | 14 | 13078.73 | 3472.38 | 61161.77 | 6601.71 |
| hexanedioic acid | 14 | 455.68 | 52.65 | 2186.71 | 201.60 |
| hexanoic acid | 10 | 2827.19 | 84.59 | 13415.56 | 995.74 |
| hexatriacontane | 9 | 96.29 | 8.77 | 518.91 | 31.33 |
| indeno(1,2,3-cd)pyrene | 15 | 436.90 | 5.17 | 3699.12 | 77.20 |
| isoeugenol | 10 | 1812.38 | 363.57 | 6155.78 | 1268.15 |
| isopimaric acid | 12 | 489.70 | 13.30 | 1204.98 | 367.35 |
| levoglucosan | 15 | 119856.68 | 12505.33 | 320299.65 | 95405.10 |
| linoleic acid | 11 | 744.41 | 17.66 | 1402.49 | 775.72 |
| linolenic acid | 13 | 225.81 | 10.14 | 481.95 | 170.81 |
| methyl flourene | 10 | 209.85 | 4.95 | 1111.99 | 44.68 |
| methyl phthalic acid | 15 | 113.01 | 29.05 | 357.80 | 75.31 |
| naphthalene | 10 | 85.01 | 1.66 | 591.93 | 31.20 |
| nonacosane | 13 | 319.67 | 14.71 | 835.73 | 163.26 |
| nonadecane | 13 | 174.44 | 8.86 | 500.37 | 151.11 |
| nonadecyl cycloheaxane | 6 | 34.22 | 6.20 | 54.26 | 32.81 |
| nonanedioic acid | 14 | 963.59 | 182.33 | 3026.44 | 785.32 |

| | | | | | |
|---|---|---|---|---|---|
| octacosane | 7 | 288.01 | 49.09 | 736.70 | 279.44 |
| octadecane | 12 | 65.30 | 12.42 | 188.27 | 55.52 |
| octadecanoic acid | 14 | 10926.94 | 1928.61 | 55466.81 | 5184.66 |
| octanendioic acid | 14 | 299.63 | 62.04 | 1011.32 | 216.07 |
| octanoic acid | 16 | 753.49 | 40.75 | 3692.13 | 486.49 |
| octatriacontane | 4 | 58.08 | 22.03 | 147.39 | 31.45 |
| oleic acid | 14 | 731.91 | 12.96 | 1672.13 | 559.79 |
| pentacosane | 14 | 191.54 | 35.42 | 610.34 | 167.82 |
| pentadecane | 9 | 929.93 | 51.55 | 2962.45 | 420.38 |
| pentadecylcyclohexane | 9 | 47.80 | 6.43 | 145.82 | 29.72 |
| pentanedioic acid | 15 | 1403.60 | 255.56 | 3638.27 | 1228.62 |
| pentatriacontane | 11 | 88.78 | 10.14 | 425.34 | 31.48 |
| perylene | 14 | 112.74 | 1.10 | 937.08 | 24.56 |
| phenanthrene | 15 | 1722.41 | 30.29 | 10425.53 | 414.08 |
| pinonic acid | 10 | 531.87 | 14.64 | 2019.62 | 93.26 |
| propylguaiacol | 2 | 149.56 | 146.59 | 152.53 | 149.56 |
| pyrene | 15 | 3024.19 | 154.97 | 14632.47 | 1056.82 |
| retene | 15 | 1984.62 | 70.40 | 5311.90 | 1314.23 |
| syringealdehyde | 15 | 12500.04 | 1043.16 | 38283.93 | 8658.35 |
| tetracosane | 15 | 299.73 | 3.17 | 1305.56 | 231.03 |
| tetracosanoic acid | 14 | 983.38 | 40.87 | 2636.94 | 859.56 |
| tetradecane | 5 | 385.06 | 27.20 | 724.17 | 518.83 |
| tetradecanoic acid | 14 | 1468.22 | 319.37 | 6961.07 | 706.18 |
| tetratriacontane | 11 | 103.08 | 9.44 | 456.80 | 29.83 |
| triacontane | 14 | 182.65 | 31.79 | 785.26 | 97.33 |
| triacontanoic acid | 6 | 2626.12 | 547.13 | 6394.53 | 2146.70 |
| tricosane | 15 | 216.74 | 11.31 | 542.27 | 210.92 |
| tridecane | 5 | 326.82 | 41.29 | 598.93 | 319.62 |
| tritriacontane | 13 | 134.35 | 14.42 | 624.64 | 56.42 |
| undecane | 2 | 148.50 | 92.86 | 204.14 | 148.50 |
| vanillin | 13 | 5047.37 | 62.59 | 14267.25 | 5188.31 |
| octacosanoic acid | 5 | 923.57 | 307.81 | 1793.44 | 923.51 |

**Emission factor calculations**

For each test run, emission factors for the target pollutants were calculated in terms of mass of fuel burned. The mass emission ($M_x$) for a set burn time $t$ for each target compound is calculated in the dilution tunnel as follows:

$$M_x = \sum_t (C_{x,t} - C_{x,a}) \cdot V \cdot t \quad (1)$$

Where:

$C_{x,t}$ = the concentration (mass/volume) of the target compound $x$ in the dilution duct,

$C_{x,a}$ = the ambient concentration (mass/volume) of the target compound $x$, and

$V$ = the volumetric flow rate (volume/time) in the dilution tunnel at time $t$.

Most concentrations were determined on a test average basis. The ambient concentration for total PM utilized an ambient air sample extracted beside the boiler during 100% load operation firing switchgrass pellets. Remaining concentrations were corrected with concentrations determined from dilution duct sampling with no firing (i.e. cold) of the boiler.

Volumetric flow rate was determined by multiplying the average of the dilution duct velocity measured before and after each test by the cross-sectional area of the dilution duct at the point measured. The 10-inch duct had a cross-sectional area of 0.0506 $m^2$ (0.545 $ft^2$).

Emission factors were calculated and reported per mass of fuel burned.

The emission factor per mass of fuel burned ($EF_{m,x}$) is calculated as:

$$EF_{m,x} = \frac{M_x}{M_f} \quad (2)$$

**Dilution Factor**

Measurements made at the stack or after secondary dilution were corrected to dilution duct concentrations using a dilution factor. Due to failure in the CEM measurements, estimates were made for $CO_2$ concentrations in the dilution duct for use in dilution factor calculations. The average $CO_2$ in the dilution duct was estimated based on the mass of fuel burned in each test ($M_f$), the carbon concentration in the fuel from the ultimate analysis, and the volumetric flow rate determined for the test. The calculation estimates the volume of $CO_2$ emitted divided by the volume of flow in the dilution duct corrected for ambient $CO_2$ concentration:

$$C_{CO2,t} = \frac{M_f \cdot \%C}{100 \cdot t} \cdot \frac{1}{MW_{Carbon}} \cdot \frac{SV}{V} + C_{CO2,a} \quad (3)$$

Where:

$C_{CO2,t}$ = the $CO_2$ concentration in the duct,

$C_{CO2,a}$ = the ambient concentration of $CO_2$,

$\%C$ = the carbon concentration in the fuel (weight percent),

$MW_{Carbon}$ = the molecular weight of carbon, and

$SV$ = the specific volume of an ideal gas at 20 °C and 1 atmosphere.

Concentrations measured at the stack or on secondary dilution were corrected to dilution duct concentrations for emission calculations.

The dilution factor at the stack were based on the estimated dilution duct $CO_2$ concentration and the average stack $CO_2$ concentration:

$$DF = \frac{C_{CO2,t} - C_{CO2,a}}{Stack\ CO2} \quad (4)$$

The dilution used for secondary dilution was performed with nitrogen containing minimal $CO_2$:

$$DF = \frac{C_{CO2,t}}{SD_{CO2,t}} \quad (5)$$

Where:

$SD_{CO2,t}$ = Secondary dilution $CO_2$ concentration during sample interval

Because the nature of operations and the short-term nature of the measurements taken on secondary dilution, each measurement was corrected using time specific dilution factors. Due to the quality of duct $CO_2$ measurement, time specific dilution duct $CO_2$ concentrations were estimated using the average dilution duct $CO_2$ measurements for the specific sampling time, average dilution duct $CO_2$ concentrations for total test, and the average fuel based estimate of dilution duct $CO_2$ concentrations calculated as above:

$$C_{CO2,t} = \frac{Fuel\ Based\ Average\ CO2}{CEM\ Test\ average\ CO2} \cdot CEM\ sample\ average\ CO2 \quad (6)$$

## References


1. Fine, P. M.; Cass, G. R.; Simoneit, B. R. T., Chemical Characterization of Fine Particle Emissions from Fireplace Combustion of Woods Grown in the Northeastern United States. *Environ. Sci. Technol.* **2001**, 35 (13), 2665-2675.
2. McDonald, J. D.; Zielinska, B.; Fujita, E. M.; Sagebiel, J. C.; Chow, J. C.; Watson, J. G., Fine Particle and Gaseous Emission Rates from Residential Wood Combustion. *Environ. Sci. Technol.* **2000,** *34* (11), 2080-2091.
3. Kinsey, J. S.; Touati, A.; Yelverton, T. L. B.; Aurell, J.; Cho, S.-H.; Linak, W. P.; Gullett, B. K., Emissions characterization of residential wood-fired hydronic heater technologies. *Atmos. Environ.* **2012,** *63*, 239-249.
4. Tomsej, T.; Horak, J.; Tomsejova, S.; Krpec, K.; Klanova, J.; Dej, M.; Hopan, F., The impact of co-combustion of polyethylene plastics and wood in a small residential boiler on emissions of gaseous pollutants, particulate matter, PAHs and 1,3,5- triphenylbenzene. *Chemosphere* **2018,** *196*, 18-24.
5. Horak, J.; Kubonova, L.; Krpec, K.; Hopan, F.; Kubesa, P.; Motyka, O.; Laciok, V.; Dej, M.; Ochodek, T.; Placha, D., PAH emissions from old and new types of domestic hot water boilers. *Environ. Pollution* **2017,** *225*, 31-39.
6. Krugly, E.; Martuzevicius, D.; Puida, E.; Buinevicius, K.; Stasiulaitiene, I.; Radziuniene, I.; Minikauskas, A.; Kliucininkas, L., Characterization of Gaseous- and Particle-Phase Emissions from the Combustion of Biomass-Residue-Derived Fuels in a Small Residential Boiler. *Energy Fuels* **2014,** *28* (8), 5057-5066.
7. Orasche, J.; Seidel, T.; Hartmann, H.; Schnelle-Kreis, J.; Chow, J. C.; Ruppert, H.; Zimmermann, R., Comparison of Emissions from Wood Combustion. Part 1: Emission Factors and Characteristics from Different Small-Scale Residential Heating Appliances Considering Particulate Matter and Polycyclic Aromatic Hydrocarbon (PAH)-Related Toxicological Potential of Particle-Bound Organic Species. *Energy Fuels* **2012,** *26* (11), 6695-6704.


Schematic 1. Pellet boiler testing facility and BB photo; Figure S1. Study-wide distributions, quantiles, and summary statistics for OC-EC concentrations in the PHBB emissions. In the outlier box plot, the confidence diamond contains the mean and the 95% confidence interval about the mean. The red bracket outside the box is referred to as the 'shortest half' or the densest 50% of the observations; Figure S2. Individual mean SVOC concentrations in PM emitted from boiler testing. Concentrations are given in units of  g/g OC. Whiskers indicate the concentration range. The y-axis is

log scale. Symbols and colors are coded by compound class. The descriptive statistics for each individual compound is provided in Table S2; Figure S3. PM emissions factors (g/kg) for a variety of biomass burning studies (n= 87) examining residential (R) fireplace and woodstove and commercial and residential boiler (B) appliances. Fuel type is coded by symbol type and color. Fine et al. 20011 and McDonald et al. 20002 investigated residential fireplaces and woodstoves. Kinsey et al. 20123 tested outdoor wood hydronic heaters (38 kW-50 kW). Tomsej et al. 20184 examined a 20 kW commercial boiler. Horak et al. 20175 looked at a variety of 20 kW boiler types, including a gasifier, and auto-, over-fire, and down-draft boilers. Krugly et al. 20146 looked at a 13 kW boiler, and Orasche et al. 20127 studied a boiler and residential stove. The focus is only on studies that report EFs in units of w/w. Horak et al. 20175 boiler power is estimated based on nominal power output. Orashe et al. 20127 values are converted using their provided fuel calorific value. All values are presented as averages and represent a wide variety of power outputs. The solid line at 5.2 g/kg is the study-wide mean; the median was 2.5 g/kg; Figure S4. Literature comparison of organic and elemental carbon emission factors (g/kg, Panel A) and OC/EC emissions ratios (Panel B) for PM emitted from biomass burning in residential fireplaces and boiler appliances. Values for the current study are the studies in Figure 3 means by pellet type. In panel B, fuel type is coded by symbol type and color; Figure S5. Calculated mean (with standard error) and median values of individual PAH concentrations as determined using the literature values provided in Figure 4. Values are grouped by fuel type; Table S1. Fuel Analysis Results; Table S2. Descriptive statistics for individual SVOC concentrations in the PM emitted from boiler testing. All experiments are combined. Concentrations are given in units of g/g OC.
